# Sensor Saturation Compensated Smoothing Algorithm for Inertial Sensor Based Motion Tracking

**DOI:** 10.3390/s140508167

**Published:** 2014-05-06

**Authors:** Quoc Khanh Dang, Young Soo Suh

**Affiliations:** Department of Electrical Engineering, University of Ulsan, Mugeo-dong, Namgu, Ulsan 680-749, Korea; E-Mail: khanhdq86@yahoo.com

**Keywords:** sensor saturation compensation, smoothing algorithm

## Abstract

In this paper, a smoothing algorithm for compensating inertial sensor saturation is proposed. The sensor saturation happens when a sensor measures a value that is larger than its dynamic range. This can lead to a considerable accumulated error. To compensate the lost information in saturated sensor data, we propose a smoothing algorithm in which the saturation compensation is formulated as an optimization problem. Based on a standard smoothing algorithm with zero velocity intervals, two saturation estimation methods were proposed. Simulation and experiments prove that the proposed methods are effective in compensating the sensor saturation.

## 1. Introduction

In motion tracking, there are many ways to estimate the trajectory of a moving object. Moving objects can be tracked accurately by using visual devices such as camera systems [1,2]. In [1], with 40 landmarks attached on the body, Hong *et al.* used the Eagle Digital motion capture system with seven cameras to analyze 14 angles and one ratio of gait features. In [2], Lee and Grimson investigated person identification and gender classification based on moments computed from the silhouette of walking people. However, these camera systems are limited in their setup ranges and sometimes have high implementation costs. Moreover, the angle views of the cameras are also limited and they are easily affected by illumination. Due to these reasons, for long distance or outdoor measurements, motion tracking based on camera systems seems to be a difficult task.

To avoid the mentioned disadvantages, inertial measurement units (IMU) can be used instead as wearable devices. IMUs are widely used due to their small size and low cost. With the development of the technology, IMUs are now becoming more accurate. In [3], Tadano *et al.* proposed a method using quaternion calculations from seven sensor units consisting of a tri-axial acceleration and gyro sensors. The quaternions, which are computed from the sensors attached on limbs and waist, are used in a gait wire frame model to generate the gait animation. To increase the accuracy, the IMUs are used with other aiding devices such as cameras in [4,5] or force sensors in [6].

However, IMUs still have their own limitations such as susceptibility to noise and limited dynamic range. The accuracy of inertial sensor-based estimation can be improved by using zero velocity updates as in [7], or taking advantage of the relative position and attitude of multiple sensors [8,9]. In [7], a robot arm control with an automatic calibration function based on inertial sensors is proposed. The authors state that the drift of the sensors is clearly removed by applying zero velocity updates. In [8], Helten *et al.* introduce another way which takes into account the relative position and attitude of multiple sensors to improve the accuracy of human motion estimation. Using the same method, Tao *et al.* [9] estimate limb movement. They also use the limb biomechanical model characteristics to provide constraints for sensors' relations. However, the other drawback of the inertial sensor, the saturation, has not been considered. Saturation is a state in which the signal that needs to be measured is larger than the dynamic range of the sensor. When that happens, the output of the sensor becomes the limiting value of the sensor range. This induces a considerable error between the true and estimated values during motion tracking. In this paper, we propose two methods for estimating the sensor saturation. Assuming that the motion is between not moving intervals (called zero velocity intervals), we formulate a saturation estimation problem as an optimization problem by modifying a smoother used in [10,11]. In the proposed methods, some state constraints mentioned in [12] could be added to increase the accuracy of the algorithm.

The paper is organized in five main sections and a conclusion. Section 2 points out the problem formulation. In Section 3, a standard smoothing algorithm with zero velocity intervals is described in detail. Sections 4 and 5 propose some methods for sensor saturation estimation. Some experiments to verify the proposed methods are given in Section 6. The last section concludes the paper.

## 2. Problem Formulation

We consider a moving object case where an IMU is attached on the object. There are two coordinate systems in this paper: the navigation coordinate frame and the body coordinate frame. The *z* axis of the navigation coordinate frame coincides with the local vertical. The choice of *x* axis is arbitrary. The body coordinate frame is defined as a frame with three axes coinciding with the three axes of the inertial measurement unit. The subscripts *b* (body) and *n* (navigation) are used to emphasize that a vector or matrix belongs to the body or navigation coordinate frame, respectively.

Our goal is to estimate attitude (expressed using the quaternion), position and velocity of the object from the sensor data. Let *q*=[*q*_0_ *q*_1_ *q*_2_ *q*_3_]∈*R*^4^ be a quaternion representing the rotation relationship between the navigation coordinate frame and the body coordinate frame. Let *C*(*q*) ∈ *R*^3×3^ be the rotation matrix corresponding to the quaternion *q* [13], and *r* ∈ *R*^3^ and *v* ∈ *R*^3^ be the position and velocity of the object, respectively. We have the following basic equations [14]:

(1) $$\begin{array}{l} \dot{q}=\frac{1}{2}\Omega \left(\omega_{b}\right)q\\ {\dot{v}}_{n}=a_{n}=C^{T}\left(q\right)a_{b}\\ {\dot{r}}_{n}=v_{n} \end{array}$$

where *a_n_* ∈ *R*^3^ and *a_b_* ∈ *R*^3^ are the acceleration made by forces other than gravitational field in the navigation coordinate frame and body coordinate frame, respectively. The symbol Ω is defined by:

$$\Omega \left(\omega \right)≜\left[\begin{array}{cccc} 0 & -\omega_{x} & -\omega_{y} & -\omega_{z}\\ \omega_{x} & 0 & \omega_{z} & -\omega_{y}\\ \omega_{y} & -\omega_{z} & 0 & \omega_{x}\\ \omega_{z} & \omega_{y} & -\omega_{x} & 0 \end{array}\right]$$

where *ω_b_*=[*ω_x_ ω_y_ ω_z_*]*^T^* is the body angular rate.

The inertial measurement unit used in this paper consists of three axis gyroscopes and accelerometers. Let *y_g_* ∈ *R*^3^ be the gyroscope output and *y_a_* ∈ *R*^3^ be the accelerometer output. They satisfy the following relationship [15]:

(2) $$\begin{array}{l} y_{g}=\omega_{b}+n_{g}\\ y_{a}=a_{b}+C\left(q\right)\bar{g}+n_{a} \end{array}$$

where *n_g_* and *n_a_* are zero mean white Gaussian sensor noises with covariances $R_{g}=E\left\{n_{g}n_{g}^{T}\right\}$ and $R_{a}=E\left\{n_{a}n_{a}^{T}\right\}$, and *ḡ*=[0 0 g]*^T^* The symbol *g* denotes the gravitational acceleration. It is assumed the sensor bias is already compensated using the standard calibration algorithm [16].

In summary, our goal is to estimate the quaternion, velocity and position of a moving object using the accelerometer and gyroscope data where there could be sensor saturation. Since a smoother is used instead of a filter, we note that the proposed method is offline analysis of attitude and position.

## 3. Standard Smoothing Algorithm with Zero Velocity Intervals

In this section, a standard smoothing algorithm is formulated in the quadratic optimization problem. A general method of formulating the smoothing problem in the optimization problem is given in [10]. In this section, we apply the result in [10] to an attitude and position estimation problem, where there are zero velocity intervals. Sensor saturation compensation is not considered in this section and will be discussed in Section 4.

**Figure 1.**
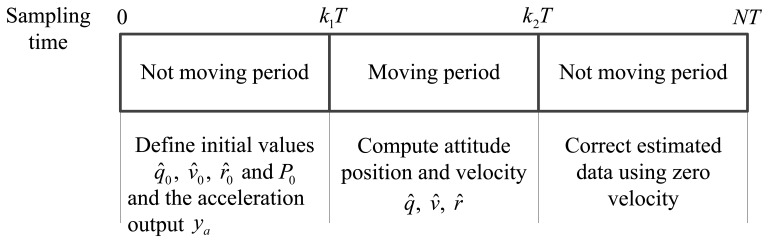
Standard inertial navigation algorithm with zero velocity correction.

We assume that the motion is a short movement consisting of a moving interval and two not moving intervals (see Figure 1). This type of movement can be found in walking [4,5], golf swing [17] and so on. The sensor data are sampled with the sampling period *T*. It is assumed that there are total *N* sampling sensor data and the moving interval starts at *k*_1_*T* and stop at *k*_2_*T* (*k*_1_ < *k*_2_ < *N*). The rest are the zero velocity intervals. We use the subscript *k* to describe a variable is expressed in the discrete time *k*. For example, a gyroscope output data at time *k* is denoted by *y_g,k_*.

Using the gyroscope output *y_g_* in Equation (1), the *q*, *v* and *r* can be estimated by the following equation (“∧” denotes for estimation):

(3) $$\begin{array}{l} \dot{\hat{q}}=\frac{1}{2}\Omega \left(y_{g}\right)\hat{q}\\ {\dot{\hat{v}}}_{n}=a_{n}=C^{T}\left(\hat{q}\right)y_{a}-\bar{g}\\ {\dot{\hat{r}}}_{n}={\hat{v}}_{n} \end{array}$$

The initial values of position *r̂*_0_ and velocity *v̂*_0_ are assumed to be zero due to the fact that the object is not moving at the not moving period and the origin of the navigation coordinate frame coincides with the starting point of the object. The initial quaternion *q̂*_0_ is obtained from the accelerometer data using the following (note that *a_b_*=0 during the zero velocity intervals):

$$y_{a}=C\left({\hat{q}}_{0}\right)\bar{g}$$

The heading in *q̂*_0_ is not determined and can be chosen arbitrarily. Since the noise terms are included in *y_g_* and *y_a_* in Equation (3), *q̂*, *v̂* and *r̂* are different from the true *q*, *v* and *r*. The error in *q̂*, *v̂* and *r̂* are estimated using a smoothing algorithm. Thus a smoothing algorithm is not used for directly estimating *q*, *v* and *r* but for estimating errors in *q̂*, *v̂* and *r̂*. Once the errors are estimated, we can update *q̂*, *v̂* and *r̂* to obtain more accurate estimates. A multiplicative error is used for the error in *q̂* [18]. A small error in *q̂* is denoted by *q_e_*. *q_e_* is assumed to satisfy the following:

(4) $$q=\hat{q}⊗q_{e}$$

or in matrix expression:

(5) $$C\left(q\right)=C\left(q_{e}\right)C\left(\hat{q}\right)$$

Since we assume *q_e_* is small, it can be approximated by $q_{e}\approx \left[\begin{array}{c} 1\\ {\bar{q}}_{e} \end{array}\right]\in \left[\begin{array}{c} R\\ R^{3} \end{array}\right]$, and ⨂ is the quaternion multiplication. Therefore, the quaternion error in *q*∈*R*^4^ is represented by *q̄_e_*=[*q̄_e_*,_1_ *q̄_e_*,_2_ *q̄_e_*,_3_]*^T^*∈*R*^3^. Assuming *q̄_e_* is small, *C*(*q_e_*) can be approximated by the following [19]:

(6) $$C\left(q_{e}\right)\approx \left[\begin{array}{ccc} 1 & 2{\bar{q}}_{e,3} & -2{\bar{q}}_{e,2}\\ -{\bar{q}}_{e,3} & 1 & 2{\bar{q}}_{e,1}\\ 2{\bar{q}}_{e,2} & -2{\bar{q}}_{e,1} & 1 \end{array}\right]=I-2\left[{\bar{q}}_{e}\times \right]$$

where [*p*×] (*p*=[*p*_1_ *p*_2_ *p*_3_]*^T^* ∈ *R*^3^) is defined by:

$$\left[p\times \right]≜\left[\begin{array}{ccc} 0 & -p_{3} & p_{2}\\ p_{3} & 0 & -p_{1}\\ -p_{2} & p_{1} & 0 \end{array}\right]$$

For the error in *v̄* and *r̄*, an additive error model is used. We use symbols *v_e_* and *r_e_* to denote the velocity error and position error, respectively:

(7) $$\begin{array}{c} v_{e}≜v_{n}-\hat{v}\\ r_{e}≜r_{n}-\hat{r} \end{array}$$

The estimation error in *q̄*, *v̄* and *r̄* can be expressed using the following state:

$$x\left(t\right)≜\left[\begin{array}{c} {\bar{q}}_{e}\\ r_{e}\\ v_{e} \end{array}\right]\in R^{9}$$

This *x*(*t*) is estimated using a smoother. To do that, we derive a differential equation for *x*(*t*). From the assumption that *q̄_e_* and *ω*−*y_g_* is small, we obtain the follow (see [19] for the derivation):

(8) $$\dot{x}\left(t\right)=Ax\left(t\right)+\left[\begin{array}{c} -\frac{1}{2}n_{g}\\ 0\\ -C^{T}\left(\hat{q}\right)n_{a} \end{array}\right]$$

where:

$$A≜\left[\begin{array}{ccc} \left[-y_{g}\times \right] & 0 & 0\\ 0 & 0 & I\\ -2C^{T}\left(\hat{q}\right)\left[y_{a}\times \right] & 0 & 0 \end{array}\right]$$

and:

$$E\left\{[\begin{array}{c} -\frac{1}{2}n_{g}\\ 0\\ -C^{T}\left(\hat{q}\right)n_{a} \end{array}]\;{\left[\begin{array}{c} -\frac{1}{2}n_{g}\\ 0\\ -C^{T}\left(\hat{q}\right)n_{a} \end{array}\right]}^{T}\right\}=\left[\begin{array}{ccc} 0.25R_{g} & 0 & 0\\ 0 & 0 & 0\\ 0 & 0 & C^{T}\left(\hat{q}\right)R_{a}C\left(\hat{q}\right) \end{array}\right]$$

Since the sensor sampling period is *T*, Equation (8) is discretized with the sampling period *T* [20]:

(9) $$x_{k+1}=A_{d,k}x_{k}+w_{k}$$

where *A_d,k_*≈exp(*A*(*kT*)*T*) and $Q_{d,k}=E\left[w_{k}w_{k}^{T}\right].$

Since no external sensors other than the inertial sensor are used, there is no physical measurement during the motion. However, the fact that the velocity is zero during the not moving period can be used as a virtual measurement. In motion analysis, it is assumed that the object is not moving when the gyroscope and the variation of the accelerometer are smaller than threshold values for some specified time. Thus there will be a chance that a moving interval is detected as a zero velocity interval. To reflect this fact, a small noise *n_v,k_* is added in the following equation:

(10) $$\begin{array}{ll} 0-\hat{v} & =v_{e}+n_{v}\\ & =\left[\begin{array}{ccc} 0_{3} & 0_{3} & I_{3} \end{array}\right]x+n_{v} \end{array}$$

The noise *n_v,k_* at time *k* is modeled as a Gaussian white noise with the covariance $R_{v,k}=E\left\{n_{v,k}n_{v,k}^{T}\right\}$. Equation 10 can be rewritten in form of following expression in discrete time at time *k*:

(11) $$z_{k}=H_{k}x_{k}+n_{v,k}$$

where *z_k_*=[0−*v̂_k_*] and *H_k_*=[0_3_ 0_3_ *I*_3_]. Equation (10) can be used during the not moving interval.

We introduce a set *Z_m_* which consist of the discrete time indices belonging to the zero velocity intervals. That is, if *k*∈*Z_m_*, then we can use the Equation (11).

A smoother problem to estimate x_k_ can be formulated as the following optimization problem [10,11]:

Find *x_k_* and *w_k_* for 0≤*k*≤*N* that minimize:

(12) $$J\left(x_{k},w_{k}\right)=\frac{1}{2}\sum_{k=1}^{N-1}w_{k}^{T}Q_{d,k}^{-1}w_{k}+\frac{1}{2}\sum_{k\in Z_{m}}{\left(z_{k}-H_{k}x_{k}\right)}^{T}R_{k}^{-1}\left(z_{k}-H_{k}x_{k}\right)+\frac{1}{2}{\left(x_{0}-{\hat{x}}_{0}\right)}^{T}P_{0}^{-1}\left(x_{0}-{\hat{x}}_{0}\right)$$

subject to *x_k+_*_1_=*A_d,k_x_k_*+*w_k_*. It is assumed that the initial value *x̂*_0_ and the initial covariance error *P*_0_ are given. The method to choose these initial values will be discussed Section 5. The Equation (12) is posed in the maximum a posterior form. The joint a posterior probability density of *x*_0_ and *w*_0_,…,*w_k+_*_1_ conditioned on *x̂*_0_,*y*_1,…_,*y_k_* is proportional to exp(−*J*), which implies that minimizing *J* means maximizing this probability density.

By inserting the constraint *x_k+_*_1_=*A_d,k_x_k_*+*w_k_* into Equation (12), we can remove the variable *w_k_* from the optimization problem:

(13) $$J\left(x_{k}\right)=\frac{1}{2}\sum_{k=1}^{N-1}{\left(x_{k+1}-A_{d}x_{k}\right)}^{T}Q_{d,k}^{-1}\left(x_{k+1}-A_{d}x_{k}\right)+\frac{1}{2}\sum_{k\in Z_{m}}{\left(z_{k}-H_{k}x_{k}\right)}^{T}R^{-1}\left(z_{k}-H_{k}x_{k}\right)+\frac{1}{2}{\left(x_{0}-{\hat{x}}_{0}\right)}^{T}P_{0}^{-1}\left(x_{0}-{\hat{x}}_{0}\right).$$

Let the optimization variable *x̄* be defined by $\bar{x}={\left[\begin{array}{cccc} x_{0}^{T} & x_{1}^{T} & \ldots & x_{N}^{T} \end{array}\right]}^{T}\in R^{9\left(N+1\right)\times 1}$, then the matrix form of the optimization will be:

(14) $$J\left(\bar{x}\right)=\frac{1}{2}{\bar{x}}^{T}M_{1}\bar{x}+M_{2}{\bar{x}}^{T}+M_{3}$$

where *M*_1_∈*R*^9(^*^N^*^+1)×9(^*^N^*^+1)^,*M*_2_∈*R*^1×9(^*^N^*^+1)^,*M*_3_∈*R* can be computed from Equation (13). Note that Equation (14) is a quadratic function of *x̄*, which can be computed efficiently using the quadratic optimization method [21]. Minimizing Equation (14) will provide a set of estimation error. From these values, *q̂*,*v̂* and *r̂* can be updated using Equations (4) and (7).

Let the minimum solution to the problem. Equation (14) be defined by J*. Note that J* depends on *y_g,k_*, *y_a,k_*(0≤*k*≤*N*), *q̂*_0_, *r̂*_0_, *v̂*_0_ and *P_0_*. For later use, we denote J* by a function *f* as follows:

(15) $$J^{*}=f\left(y_{g,k},y_{a,k},{\hat{q}}_{0},{\hat{r}}_{0},\;{\hat{v}}_{0},P_{0}\right)=\underset{\bar{x}}{\min}J\left(\bar{x}\right)$$

In Equation (14), constraints can be easily added to improve the accuracy. For example, in gait analysis, while walking on a plane that assumed to be parallel with the *xy* plane of the local navigation coordinate frame, we can make use of zero *z* axis position as following:

$$0-\left[\begin{array}{ccc} 0 & 0 & 1 \end{array}\right]{\hat{r}}_{k}=\left[\begin{array}{ccccccccc} 0 & 0 & 0 & 0 & 0 & 1 & 0 & 0 & 0 \end{array}\right]x_{k}+n_{r,k}$$

and the constraint of nonnegative *z* axis position is given by:

$$\left[\begin{array}{ccc} 0 & 0 & 1 \end{array}\right]{\hat{r}}_{k}+\left[\begin{array}{ccccccccc} 0 & 0 & 0 & 0 & 0 & 1 & 0 & 0 & 0 \end{array}\right]x_{k}\geq 0.$$

## 4. Sensor Saturation Estimation

**Figure 2.**
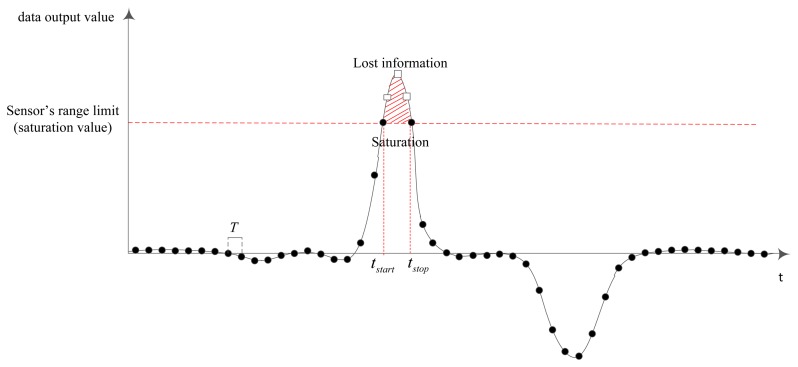
Sensor Saturation.

The sensor saturation occurs when the measured values are over the sensors' dynamic ranges (Figure 2). While it is difficult to know sensor noise values (*n_g_* and *n_a_* in Equation (7)), it is not difficult to know when the sensor saturation occurs.

Each sensor has its own range of measurement. When the measured values are larger than the measurement limit, the saturation happens. Even if the saturation happens in a short time, it could lead to a large accumulated error since the lost information is in a large magnitude data area. The data loss due to the saturation has a considerable influence to the result, especially when the integration is used in data processing.

The saturation can be avoided by using large dynamic range sensors. Usually, large measuring range (with the same sensor resolution) sensors tend to be expensive. Instead of using an expensive large range sensor, we can use a low cost one with smaller measuring limit along with applying a saturation compensation algorithm.

The saturation can happen in gyroscopes and accelerometers in three axes. Symbols *y_g,x,k_*,*y_g,y,k_*, *y_g,z,k_* are used to denote three elements in *y_g,k_*∈*R*^3^. Similarly, *y_a,x,k_*,*y_a,y,k_* and *y_a,z,k_* are used for *y_a,k_*∈*R*^3^. We use the fact that the value of the sensor output is smaller than the saturation value outside the saturation interval. In the saturation interval, we assume that the sensor output is equal to the saturation value. Let *y_g,sat_* be the saturation value of a gyroscope and *S_g,x_* be the set of gyroscope *x* saturation inertial indexes:

(16) $$\left\{ \begin{array}{l} \left|y_{g,x,k}\right|=y_{g,x,sat}\;\text{if}\;k\in S_{g,x}\\ \left|y_{g,x,k}\right|<y_{g,x,sat}\;\text{if}\;k\notin S_{g,x} \end{array}\right..$$

Similarly, we can define *S*_g,y_, *S*_g,z_, *y*_a,sat_ (saturation value of an accelerometer), *S*_a,x_, *S*_a,y_ and *S*_a,z_. Denote *δ_g_*,*_x_*,*_k_*, *δ_g_*,*_y_*,*_k_*, *δ_g_*,*_z_*,*_k_*, *δ_a_*,*_x_*,*_k_*, *δ_a_*,*_y_*,*_k_*, *δ_a_*,*_z_*,*_k_*, the compensation values of gyroscopes and accelerations in *x*,*y*,*z* axes at the time *k*, respectively. The compensated sensor value (*ȳ_g,k_* and *ȳ_a,k_*) is the sum of sensor output value and compensation value. For example, the compensated *x* axis gyroscope value is given by:

(17) $${\bar{y}}_{g,x,k}=\left\{ \begin{array}{l} y_{g,x,k}+\delta_{g,x,k}\;\text{if}\;k\in S_{g,x}\\ y_{g,x,k}\;\text{if}\;k\notin S_{g,x} \end{array}\right.$$

Let *δ* be set of *δ_g,x,k_*, *δ_g,y,k_*, *δ_g,z,k_*, *δ_a,x,k_*, *δ_a,y,k_* and *δ_a,z,k_* variables. For example, if *S_g,x_*={5,6,7}, *S_a,y_*={8,9,10}, and *S_g,y_*= *S_g,z_*= *S_a,x_*= *S_a,z_*=⊘ then *δ* is given by:

$$\delta ={\left[\begin{array}{cccccc} \delta_{g,x,5} & \delta_{g,x,6} & \delta_{g,x,7} & \delta_{a,y,8} & \delta_{a,y,9} & \delta_{a,y,10} \end{array}\right]}^{T}\in R^{6}.$$

**Figure 3.**
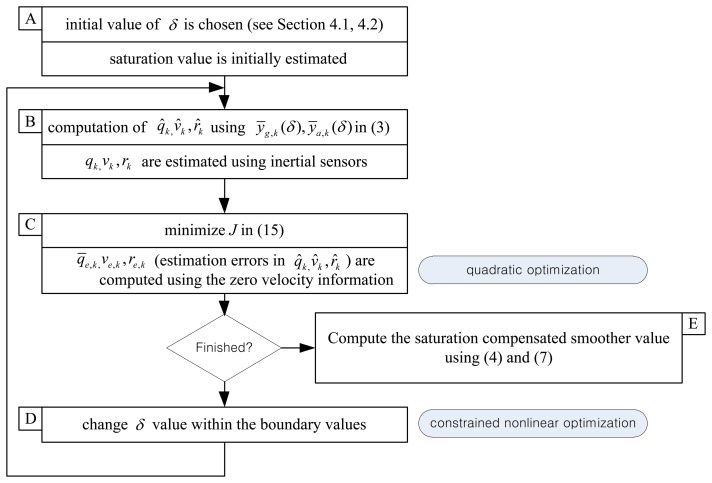
Proposed saturation compensated smoothing algorithm.

Now the standard smoother algorithm in Section 3 is modified using *ȳ_g,k_* and *ȳ_a,k_*. The flowchart of the algorithm is given in Figure 3. The first step (step A in Figure 3) of the algorithm is the initial estimation of, *δ* which is explained in Sections 4.1 and 4.2. Given *δ* values, the standard smoother algorithm (steps B and C) is applied to compute the smoother. This standard algorithm (given in Section 3) can be formulated as the quadratic optimization algorithm. How good the computed smoother is can be evaluated using the computed *J* value in Equation (15). This process (steps B and C) is repeated by changing *δ* values. The algorithm finishes if the minimum value of *J* is found. We note that *δ* optimization can be formulated as a constrained nonlinear optimization problem. Once the minimization process is done, we can compute the saturation compensated smoother values (step E).

Consider the following function (from Equation (15)):

(18) $$f\left({\bar{y}}_{g,k}\left(\delta \right),{\bar{y}}_{a,k}\left(\delta \right),{\hat{q}}_{0},{\hat{r}}_{0},{\hat{v}}_{0},P_{0}\right)$$

Assuming *q̂*_0_, *r̂*_0_, *v̂*_0_,*P*_0_,*y_g,k_* and *y_a,k_* are constant, the function *f* in Equation (18) depends on *δ*. In this section, *f* is minimized with respect to *δ*. Two different methods are proposed. The first method directly minimize *δ* with respect to all possible combination of *δ*. The second method uses the geometric structure of the sensor saturation.

### 4.1. Method 1: Direct Estimation of *δ*

In the first method, the following optimization problem is solved:

(19) $$\underset{\delta }{\min}f\left({\bar{y}}_{g,k}\left(\delta \right),{\bar{y}}_{a,k}\left(\delta \right),{\hat{q}}_{0},{\hat{r}}_{0},{\hat{v}}_{0},P_{0}\right)=\underset{\delta }{\min}\underset{\bar{x}}{\min}J\left(\bar{x}\right)$$

subject to:

(20) $$\left\{ \begin{array}{l} \delta \leq \delta_{b}\;\text{if}\;\delta \geq 0\\ \delta \geq -\delta_{b}\;\text{if}\;\delta \leq 0 \end{array}\right.$$

In Equation (20), “≤” and “≥” represent element-wise inequalities and *δ_b_* is a set of bounding positive values of elements in *δ*. Choice of *δ_b_* depends on applications. For example, in knee gyroscope data, the maximum angular velocity of a human knee varies from 213 to 1,087°/*s* [22]; in gait acceleration, the maximum foot acceleration is around 11.82 *m*/*s*^2^ for walking case [23]. Therefore, the gyroscope data upper bound can be chosen as *δ_b_* = [1,087 − *y_g,sat_* 11.82 − *y_a,sat_*]*^T^*. From Equations (16) and (17), it is easily to be seen that when *y_g_*,*y_a_*≥0, we have *δ* ≥0 because gyroscope compensated values (*ȳ_g_*, *ȳ_a_*) are larger than saturation value (*y_g,sat_*,*y_a,sat_*). The initial value of *δ* can be chosen as a set of 0 and varies in a range which satisfies the condition in Equation (20). With each set of *δ*, one value of *x̄* is obtained. The minimum value of *δ* which makes *x̄* minimizes the quadratic problem (14) is chosen.

### 4.2. Method 2: Estimation of *δ* Using Geometric Form

**Figure 4.**
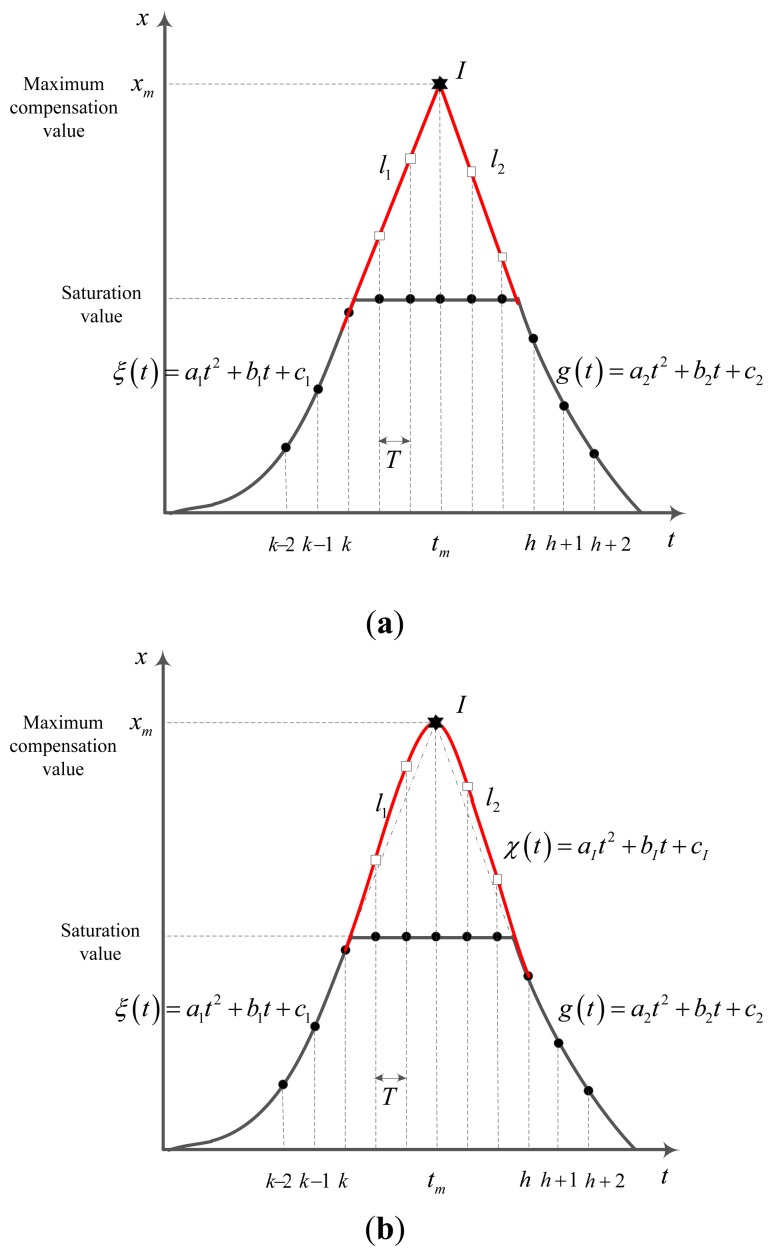
Estimation of *δ* using geometric form. (**a**) Triangle approximation; (**b**) Quadratic approximation.

In this method, the saturated sensor data region is approximated by a triangle (see Figure 4a) and quadratic function (see Figure 4b). Using three data before the saturation interval (*x_k_*_−2_, *x_k_*_−1_,*x_k_* in Figure 4a), we can obtain a quadratic function. In the next step, a line *l*_1_ starting from *x_k_* with the slope ξ'(*k*) is generated. With the same procedure (quadratic function *g*(*t*) for *x_h_*,*x_h+1_*,*x_h+2_*), we generate a line *l*_2_. *l*_1_ and *l*_2_ are the reconstruction part of sensor saturation. The intersection point of *l_1_* and *l*_2_ (at the time *t_m_*) is the maximum value (*x_m_*) for compensation. Now consider *l*_1_ is created based on (*x_k_*,*k*) and (*x_m_*,*t_m_*) points, *l*_2_ is created based on (*x_h_*,*h*) and (*x_m_*,*t_m_*) points. Changing the height of the intersection from saturation value to maximum compensation value *x_m_* we can generate several sets of basic lines *l*_1_,*l*_2_ which contain compensation values. The intersection point is defined as following:

(21) $$\begin{array}{l} t_{m}=\frac{g\left(h\right)-\xi \left(k\right)-\left(h-k\right)g^{\prime }\left(h\right)}{\xi^{\prime }\left(k\right)+g^{\prime }\left(h\right)}\\ x_{m}=\xi \left(k\right)+t_{m}\xi^{\prime }\left(t_{m}\right) \end{array}$$

The other compensation value is defined by:

(22) $$\begin{array}{c} x_{t}=\xi \left(k\right)+\frac{x_{m}-\xi \left(k\right)}{t_{m}-k}\left(t-k\right)\;\text{if}\;k<t<t_{m}\\ x_{t}=g\left(h\right)+\frac{g\left(h\right)-x_{m}}{h-t_{m}}\left(t-h\right)\;\text{if}\;t_{m}<t<h \end{array}$$

Using this method, sets of sensor's compensation values *δ* can be generated by changing the intersection point's value from saturation value to maximum compensation value. In this case, *δ* only depends on intersection point. Denote *I*(*x_I_*,*t_I_*) the intersection point. Once *I* is created, the rest compensation data will be obtained based on *l*_1_ and *l*_2_ lines using Equation (22). The optimization problem (19) will subject to *δ* value which relates to the intersection point.

With the same idea, a quadratic approximation can be used to generate the lost information (see Figure 3b. Firstly the maximum compensation value can be generated using the same procedure in triangle approximation process. After this step, a quadratic function χ(*t*) is formed up based on (*x_k_*,*t_k_*),(*x_h_*,*t_h_*) and (*x_m_*,*t_m_*) points. In saturation parts, the compensation value at time *t_k_* is χ(*t_k_*).

## 5. Sensor Saturation Compensation Algorithm with Multiple Zero Velocity Intervals

**Figure 5.**
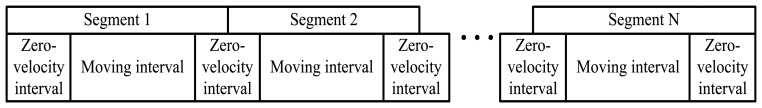
A movement with multiple zero velocity intervals.

Section 4 introduced two methods to compensate sensor saturation in a standard movement situation which contains two zero velocity intervals at the beginning and ending of a moving interval. In this section, we apply the Section 4 methods to multiple zero velocity interval movements. A movement with multiple zero velocity intervals is displayed in Figure 5. In this movement, moving intervals are interposed by zero velocity intervals. An example of this movement can be found in gait analysis in [4,5]. In the characteristic human gait, one foot is assumed to be not moving when the the center of mass is put on the corresponding leg to move the other. Therefore, during the walking process, there are zero velocity intervals separated by moving intervals for each foot.

We divide the movement into segments based on zero velocity intervals so that between two zero velocity intervals there is data from one moving period (see Figure 5). If there are saturations in the movement, we can apply the compensation methods in Section 4 to each segment. The information of the last sampling in the prior segment will be used as the initial value for the following one. The initial error covariance for each segment can be estimated using a Kalman filter with a zero velocity measurement update for previous segment. Note that for the first segment the position and velocity errors are assumed to be zero due to the fact that the initial body coordinate frame coincides with the navigation coordinate frame. The initial covariance of the first segment is also assumed to be small. Repeating the smoothening processes until the last segment, the whole smoothed data is obtained.

## 6. Simulation and Experiments

**Figure 6.**
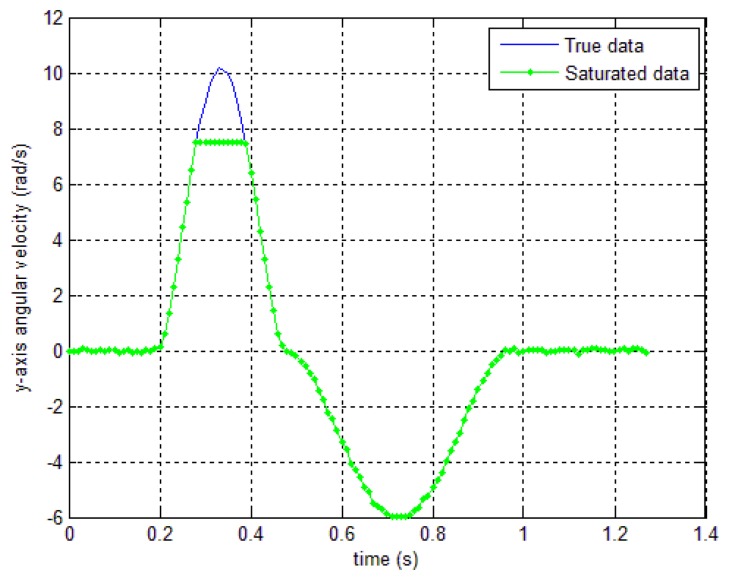
Simulation gyroscope *y_g,y_* data with and without saturation (*y_g,y,sat_*=7.5).

In this section, one simulation result and two experimental results are given to verify the proposed algorithm. First, a simulation is done to verify the proposed method. The sensor is assumed to be located at the end of a bar, which is rotated along the body *y* axis. There is a sensor's saturation in the gyroscope *y* axis data as in Figure 6. The saturated data (green “—” line) is obtained with *y_g,y,sat_*=7.5. This saturated data is used as an input data for the compensation algorithms in Section 4.

**Figure 7.**
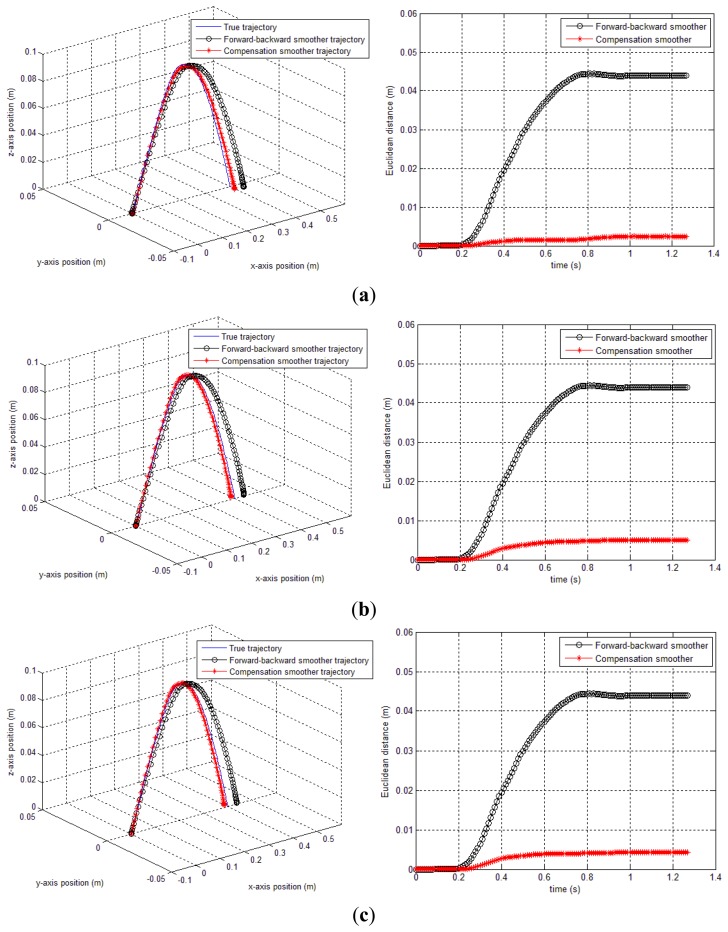
True and estimated trajectories. (**a**) method 1 result: 3D trajectory and Euclidean distance over time; (**b**) method 2 (triangle approximation): 3D trajectory and Euclidean distance over time; (**c**) method 2 (quadratic approximation): 3D trajectory and Euclidean distance over time.

In this simulation, *δ* only contains *y* axis compensation (*δ_g,y,k_*). The smoothing results (forward-backward smoother [20] and the proposed methods) are given in Figure 7 and Table 1. In case of method 1, *δ_b_* is chosen as 5, so that the performance index *J* is minimized subject to 0≤*δ*≤5. Similarly, method 2 (both triangle and quadratic approximation) is applied. In Table 1, we can see that both method 1 and 2 produce significant improvements over the conventional smoothing (forward-backward smoother) result. Among the proposed methods, method 1 gives the best result and the second best is method 2 (quadratic approximation). We note that the method 1 requires more computation than method 2 since it needs more optimization variables. As for method 2, it is not conclusive whether quadratic approximation is better than triangle approximation since triangle approximation sometimes gives better results as seen in our later experiment (see Table 2).

**Figure 8.**
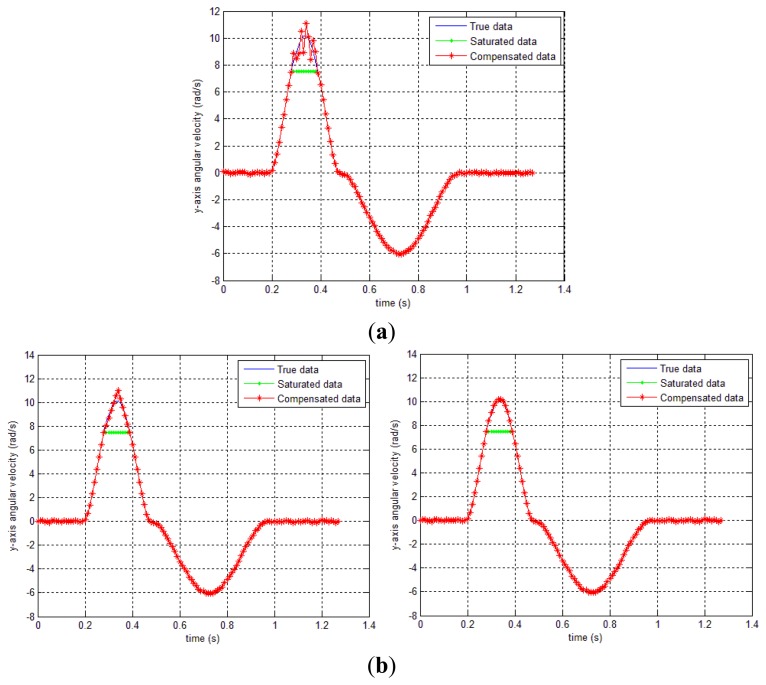
*y_g,y_* data compensation result by the proposed methods. (**a**) sensor compensation result by method 1; (**b**) sensor compensation result by method 2 (triangle and quadratic approximation).

In Figure 8, the gyroscope sensor saturation compensation results by the proposed methods are given. In all three cases, it can be seen that the sensor saturation is well estimated.

In Figure 7, the gyroscope sensor saturation compensation results by the proposed methods are given. In all three cases, it can be seen that the sensor saturation is well estimated.

**Figure 9.**
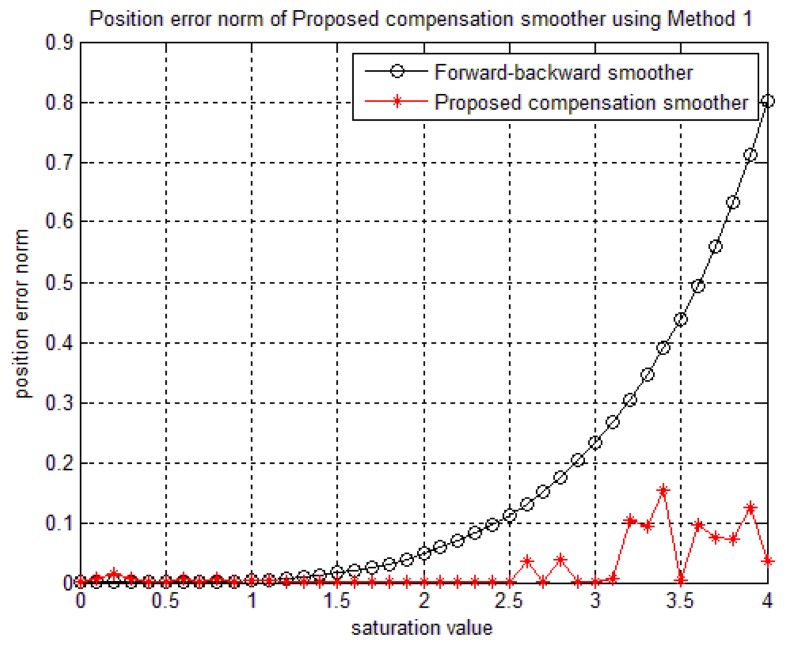
The effect of saturation value (in gyroscope data) on the method 1 smoother accuracy.

In order to verify the robustness of the proposed algorithms to the saturation, a simulation is done by checking the position norm errors while *y_g,y,sat_* value is changed. For example, if we set *y_g,y,sat_*=12 in Figure 6, there is no saturation in the sensor. On other hand, if we decrease *y_g,y,sat_* value, the sensor saturation increases. With the method 1, when the *δ_b_* is fixed and the saturation value is changed, the accuracy of the proposed smoother may be affected (if the *δ_b_* is chosen not large enough). As can be seen from Figure 9 where the *δ_b_* is chosen as 2.5, the accuracy will decrease when the compensation value that is used to compensate the saturated data is larger than. *δ_b_* However, this effect can be avoided since the saturation value is known for each sensor. Therefore, we can choose *δ_b_* as a large number compared with the sensor's saturation value.

**Figure 10.**
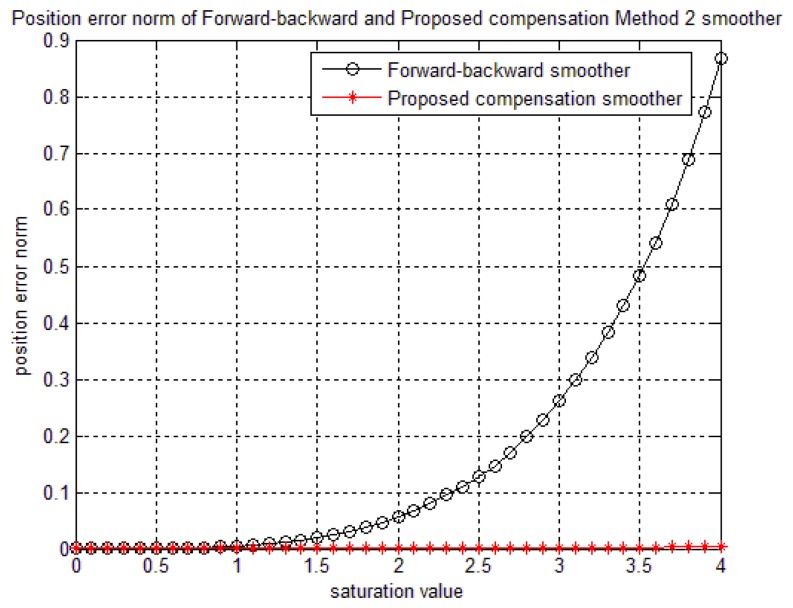
The effect of saturation value (in gyroscope data) on the method 2 smoother accuracy.

Sections 4.1 and 4.2 showed that the accuracy of the method 2 only depends on the saturation value while the accuracy of the method 1 is affected by the saturation value and the. *δ_b_* Figure 10 illustrates that the saturation is well compensated by method 2, even when the saturation value is changing. In general, compared with a forward-backward smoother, our proposed compensation smoother has better performance.

**Figure 11.**
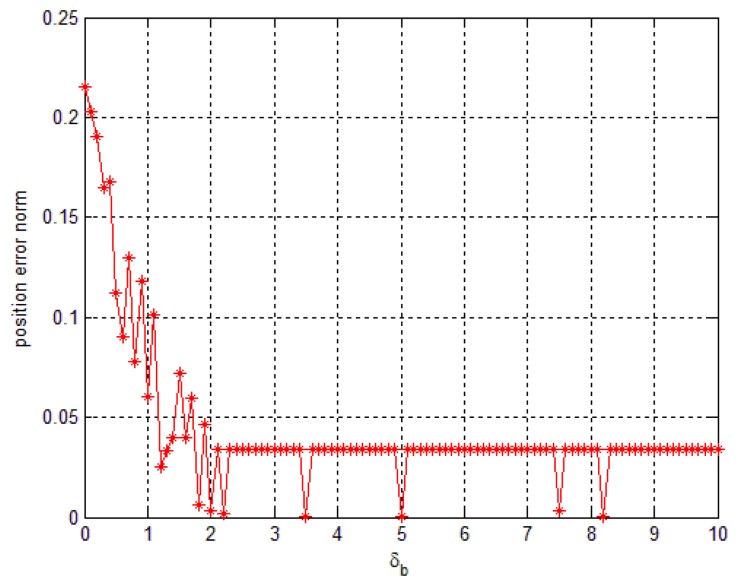
The effect of *δ_b_* on the method 1 smoother accuracy.

As mentioned above, how to choose *δ_b_* may affect the accuracy. In some applications, the *δ_b_* is known from doing statistical experiments. In the case where *δ_b_* is unknown, we can choose an arbitrarily large value. This does not affect the result as shown in Figure 11. In Figure 11, when *δ_b_* is chosen smaller than the compensation value that is used to compensate the saturated data, the error could be large. If we increase, *δ_b_* the error decreases since a larger saturation can be compensated. However, it could lead to more computations for solving the optimization Equation (19).

**Figure 12.**
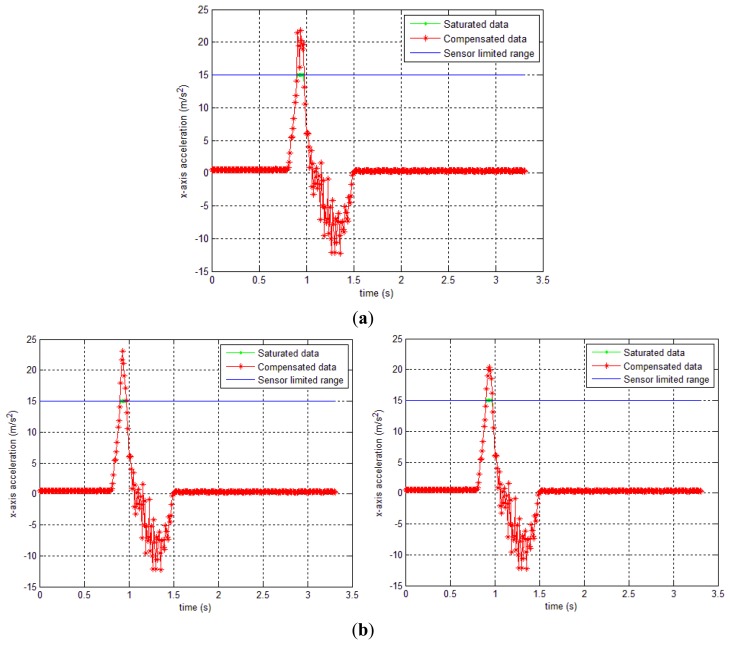
*y_a,x,k_* sensor's output and compensated data of 0.95 m straight movement experiment; (**a**) method 1 result; (**b**) method 2 results (triangle and quadratic approximation).

In the first experiment, an object, which is attached with an IMU on the top, moved in a straight line of 0.95 m so that the *x* axis of the IMU coincides with direction of movement. In this case, there exists saturation in the sensor's accelerometer in the *x* axis (*y_a,x_*). The trajectory of the object is estimated by the forward-backward smoother and our proposed compensation smoother using two methods. Figure 12 shows that the saturated *y_a,x,k_* data was compensated using the proposed compensation smoothers.

**Figure 13.**
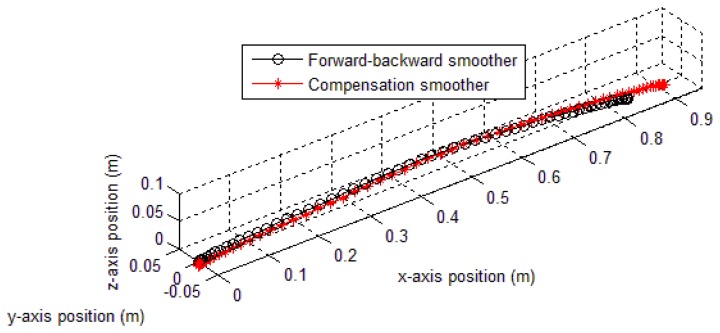
Trajectories of 0.95 m straight movement experiment (method 1 result).

The estimated position errors are given in Figure 13 (method 1 only, since the results of method 2 are similar) and Table 2. As can be seen from Figure 13, the proposed compensation smoother using method 1 gives a closer result to the true position (0.9121 m, equivalent to 4% error) than the forward-backward smoother (0.8404 m, equivalent to 11.54% error). The proposed compensation smoother using method 2 also gives a good result of 0.9084 m (4.39% error) and 0.8976 m (5.52% error) for triangle and quadratic approximations, respectively.

**Figure 14.**
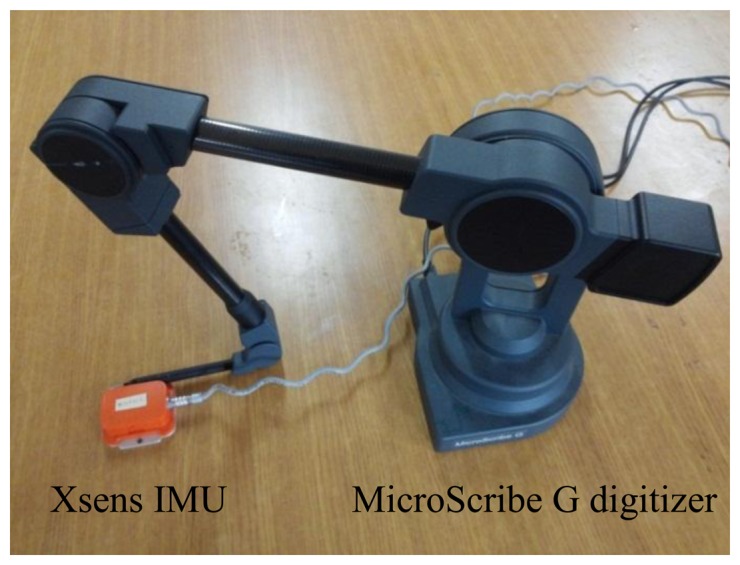
Curve movement experiment setup.

**Figure 15.**
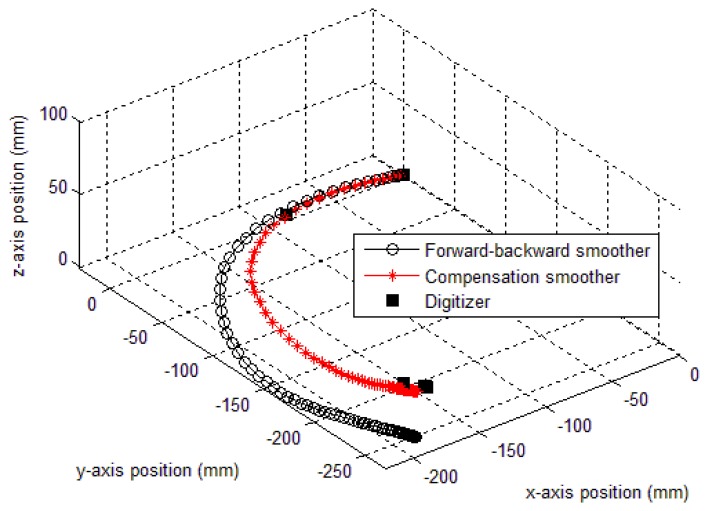
Trajectories of a curve movement experiment (method 1).

Another experiment has been done to verify the compensation feasibility of the proposed algorithm. In this experiment, an IMU is attached at the tip of a digitizer (see Figure 14). The tip was moved in a curved line. The position data of the tip was recorded by the digitizer while the trajectory of the IMU is estimated by the proposed compensation and the forward-backward smoothers, respectively. The movement was made so that there is saturation in the *y_g,z,k_* data. The estimated trajectories are compared with the true trajectory of the digitizer in Figure 15 (in millimeters). The distance between the start and stop point is used as an evaluation criterion. Based on this criterion, a table of distance errors of the estimated positions and the digitizer's data is formed, as shown in Table 3. Table 3 shows that method 1 gives a best accuracy (7.9 mm error) compared with other estimations. The forward-backward smoother has a worst estimation with a 50.4 mm error result. Moreover, the two approximation methods in method 2 provide similar results (11.8 and 10.3 mm errors).

**Figure 16.**
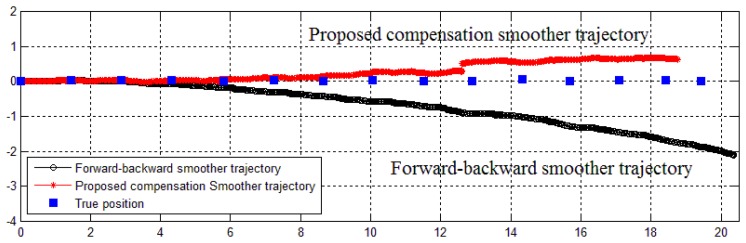
True and estimated trajectories of a walking person.

In the last experiment, we verify the compensation smoother application in multiple zero velocity intervals movement (in Section 5). In this experiment, an IMU is attached on a human foot. The volunteer was asked to walk along a straight corridor. A pen is also attached on the volunteer's shoe to mark the steps' positions on the floor. The obtained data from IMU is used to estimate the trajectory of the foot. A comparison of proposed and forward-backward smoothers trajectories is given in Figure 16. The result shows that the last position error of proposed smoother trajectory is 0.9042 m, while it is 2.3128 m for the forward-backward smoother trajectory.

## 7. Conclusions

This paper has proposed some approaches to compensate for sensor saturation. Saturation is a common problem with IMU sensors in tracking a moving object. The lost data in the saturated parts could be important due to the accumulated errors. In the paper, the authors used a standard smoothing algorithm with zero velocity intervals to compensate sensor saturation. The considered motion includes a moving interval between two zero velocity intervals. Two methods were proposed. The first method directly estimates the saturation compensation while the second one uses a geometric form to estimate the saturation. The proposed smoothing algorithm can be applied in some motions which contain many moving intervals separated by zero velocity intervals. In this case, the motion is divided into segments based on zero-velocity intervals so that between two zero velocity intervals there is one moving interval. The saturation estimation algorithm is applied in each segment from the first to the last one. To verify the feasibility of the two methods, some experiments have been done. The experiments showed that the proposed smoothing algorithm can compensate the sensor saturation and provides a smaller error than a conventional smoother (forward-backward filter). In practical applications, the sensor saturation compensation methods proposed in this paper can be used to improve the accuracy of small dynamic range sensors instead of using a large dynamic range one which usually tends to be more expensive.

## Figures and Tables

**Table 1.** Last position accuracy of different smoothers.

**Estimation Method**	**Last Position Error**	**Position Error Norm**
Forward-backward smoother	0.0458	0.1544
Proposed smoother (method 1)	0.0032	0.0009
Proposed smoother (method 2: triangle approximation)	0.0045	0.0019
Proposed smoother (method 2: quadratic approximation)	0.0036	0.0014

**Table 2.** The error of 0.95 m straight movement experiment.

**Estimation Method**	**Last Position Error**
Forward-backward smoother	0.1096
Proposed smoother (method 1)	0.0379
Proposed smoother (method 2: triangle approximation)	0.0416
Proposed smoother (method 2: quadratic approximation)	0.0524

**Table 3.** The start-stop point distance error of curve movement experiment (in mm).

**Estimation Method**	**Distance Error**
Forward-backward smoother	50.4
Proposed smoother (method 1)	7.9
Proposed smoother (method 2: triangle approximation)	11.8
Proposed smoother (method 2: quadratic approximation)	10.3
